# Genome-wide DNA methylation profiling of HPV-negative leukoplakia and gingivobuccal complex cancers

**DOI:** 10.1186/s13148-023-01510-z

**Published:** 2023-05-27

**Authors:** Mayuri Inchanalkar, Sumana Srivatsa, Srikant Ambatipudi, Priyanka G. Bhosale, Asawari Patil, Alejandro A. Schäffer, Niko Beerenwinkel, Manoj B. Mahimkar

**Affiliations:** 1 Mahimkar Lab, Cancer Research Institute (CRI), Advanced Centre for Treatment, Research and Education in Cancer (ACTREC), Tata Memorial Center, Kharghar, Navi Mumbai, Maharashtra 410210 India; 2 Homi Bhabha National Institute, Training School Complex, Anushakti Nagar, Mumbai, Maharashtra 400094 India; 3 Department of Biosystems Science and Engineering, ETH Zurich, Basel, Switzerland; 4 SIB Swiss Institute of Bioinformatics, Basel, Switzerland; 5 Achutha Menon Centre for Health Science Studies, Sree Chitra Tirunal Institute for Medical Sciences & Technology, Thiruvananthapuram, Kerala India; 6 Centre for Gene Therapy and Regenerative Medicine, Guy’s Hospital, King’s College London, Tower Wing, London, UK; 7 Department of Pathology, Tata Memorial Hospital, Tata Memorial Centre, Mumbai, India; 8 Cancer Data Science Laboratory, Center for Cancer Research, National Cancer Institute, and National Center for Biotechnology Information, National Institutes of Health, Bethesda, MD USA

**Keywords:** Leukoplakia, Gingivobuccal complex cancers, OSCC, DNA methylation, Integrative analysis, Prognosis, Differentially methylated promoters (DMPs)

## Abstract

**Background:**

Gingivobuccal complex oral squamous cell carcinoma (GBC-OSCC) is an aggressive malignancy with high mortality often preceded by premalignant lesions, including leukoplakia. Previous studies have reported genomic drivers in OSCC, but much remains to be elucidated about DNA methylation patterns across different stages of oral carcinogenesis.

**Results:**

There is a serious lack of biomarkers and clinical application of biomarkers for early detection and prognosis of gingivobuccal complex cancers. Hence, in search of novel biomarkers, we measured genome-wide DNA methylation in 22 normal oral tissues, 22 leukoplakia, and 74 GBC-OSCC tissue samples. Both leukoplakia and GBC-OSCC had distinct methylation profiles as compared to normal oral tissue samples. Aberrant DNA methylation increases during the different stages of oral carcinogenesis, from premalignant lesions to carcinoma. We identified 846 and 5111 differentially methylated promoters in leukoplakia and GBC-OSCC, respectively, with a sizable fraction shared between the two sets. Further, we identified potential biomarkers from integrative analysis in gingivobuccal complex cancers and validated them in an independent cohort. Integration of genome, epigenome, and transcriptome data revealed candidate genes with gene expression synergistically regulated by copy number and DNA methylation changes. Regularised Cox regression identified 32 genes associated with patient survival. In an independent set of samples, we validated eight genes (*FAT1, GLDC, HOXB13, CST7, CYB5A, MLLT11, GHR, LY75*) from the integrative analysis and 30 genes from previously published reports. Bisulfite pyrosequencing validated *GLDC* (*P* = 0.036)*, HOXB13* (*P* < 0.0001) promoter hypermethylation, and *FAT1* (*P* < 0.0001) hypomethylation in GBC-OSCC compared to normal controls.

**Conclusions:**

Our findings identified methylation signatures associated with leukoplakia and gingivobuccal complex cancers. The integrative analysis in GBC-OSCC identified putative biomarkers that enhance existing knowledge of oral carcinogenesis and may potentially help in risk stratification and prognosis of GBC-OSCC.

**Graphical abstract:**

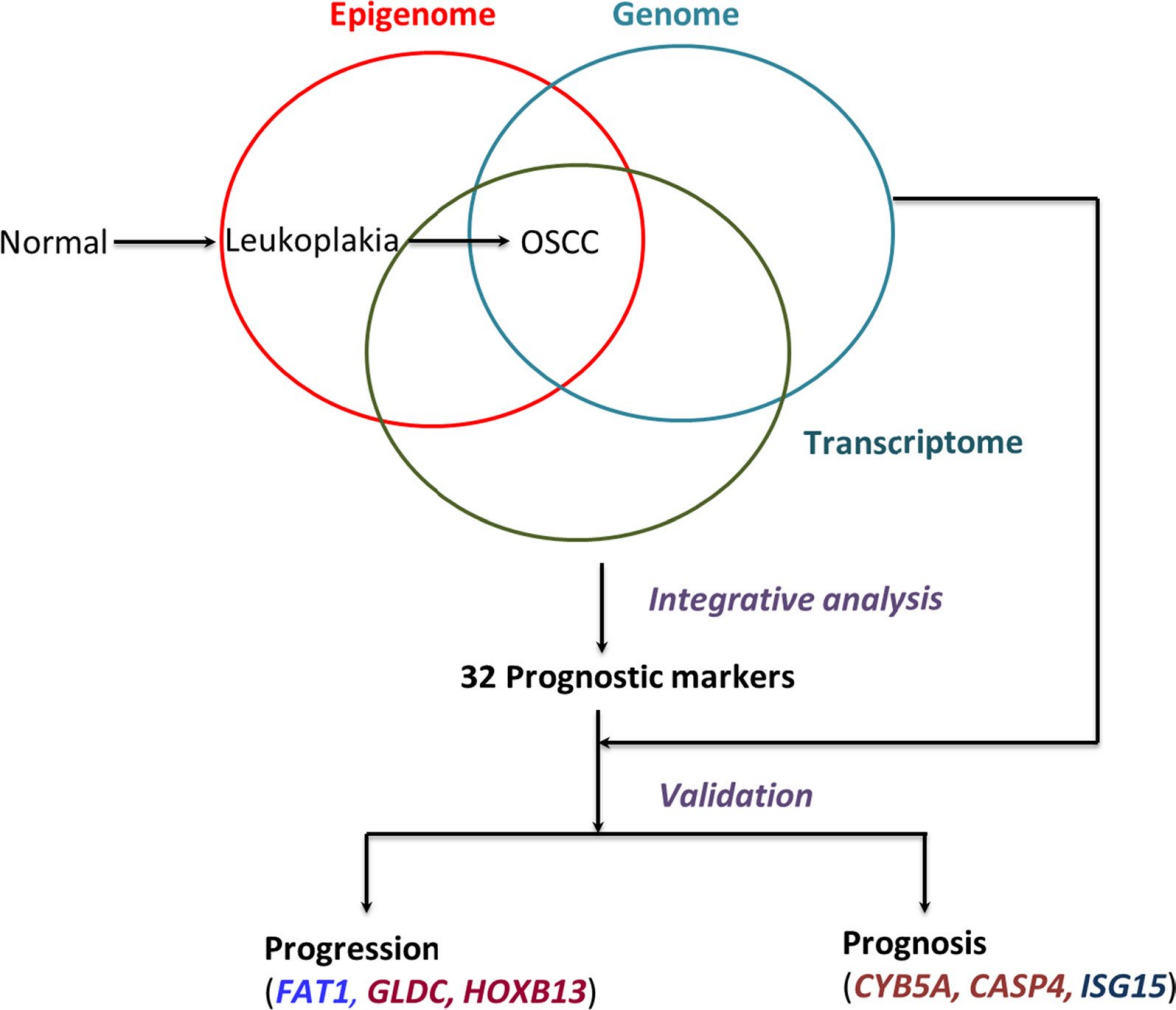

**Supplementary Information:**

The online version contains supplementary material available at 10.1186/s13148-023-01510-z.

## Background

Oral squamous cell carcinoma (OSCC) arising in the gingivobuccal complex (GBC) is the most common malignancy in India attributable to tobacco abuse [1, 2]. Oral cancers have high morbidity and mortality as they are frequently diagnosed at an advanced stage, and managing them is challenging due to early invasion. Despite advances in treatment modalities, the prognosis of OSCC remains poor due to locoregional recurrence and nodal metastasis [3]. OSCCs are often preceded by oral premalignant lesions (OPLs), now termed as oral potentially malignant disorders (OPMDs), mainly leukoplakia, which has a transformation rate to OSCC between 0.13 and 34.0% [4]. Early diagnosis and primary prevention remain the best approaches for OSCC management. Histopathology is the gold standard for diagnosis, but it is not sufficient to predict transformation potential or disease progression. Human papillomavirus (HPV) infection has emerged as an important stratification factor associated with better prognosis and treatment de-escalation. In HPV-negative OSCC, several biomarkers have been proposed, but their effectiveness is yet to be proven in the clinic. These observations justify the need for robust biomarkers for early detection of high-risk OPMDs, treatment response, and prognosis to improve OSCC management [5].

Oral carcinogenesis is a complex multifactorial molecular process that involves the accumulation of genetic and epigenetic changes leading to aberrant copy number gains and upregulation of oncogenes as well as to copy number losses and downregulation of tumor suppressor genes (TSGs). Previously, we have reported genomic and transcriptomic profiles of leukoplakia and gingivobuccal complex cancers that suggest the possible involvement of epigenetic regulation [6, 7]. DNA methylation is the most commonly studied epigenetic mechanism that regulates gene expression, and a plethora of literature is available for DNA methylation-based biomarkers [8–12] with prognostic significance [13–18]. Hypomethylation of oncogenes and hypermethylation of TSGs are responsible for disease progression [19]. Genome-wide methylation patterns have been studied extensively in OSCC (Additional file 1: Table S1). However, these studies have small sample sizes, include samples from different anatomic sites, and mostly lack representation from OPMD samples. Few studies have integrated methylation data with gene expression (GE) or copy number alteration (CNA) data. Furthermore, validation and correlation of candidate biomarkers with clinical outcomes are lacking. To overcome these lacunae, we performed a comprehensive study and identified genome-wide DNA methylation signatures, gene expression, and copy number changes associated with different stages of oral carcinogenesis. We validated selected candidate genes from these signatures and evaluated their association with clinical outcomes in GBC-OSCC patients.

## Results

### Demographic and clinicopathological details of study samples

The study group consisted of a training set for discovery and an independent validation set. Clinicopathological and demographic details of the study participants are summarised in Table 1. Additional file 1: Tables S2 and S3 show the individual characteristics of leukoplakia and gingivobuccal complex oral cancer samples with follow-up data used in this study and previous studies [6, 7]. The validation set had similar characteristics to the training set. The grading of tumors is represented in Additional file 1: Table S3, with 63% of patients having advanced stage OSCC and 38% having lymph node positivity at presentation. The majority of the patients (69%) were solely tobacco consumers, while many (19%) consumed both tobacco and alcohol. The median follow-up of the validation set was 70 months. All samples were tested for the presence of HPV and were negative for HPV-DNA, which is consistent with our previous reports on gingivobuccal cancers [20].

**Table 1:** The clinicopathological and demographic characteristics of study patients

	Training set		Validation set
	Leukoplakia (n = 22)	OSCC (n = 74)	OSCC (n = 127)
*Age at diagnosis*			
Median age	43	52.5	53
Range (IQR)	40–52.25	43–62	44–61
*Sex*			
Male	20 (90.9%)	56 (75.7%)	108 (85%)
Female	2 (9.1%)	18 (24.3%)	19 (15%)
*Pathological stage*			
Stage 1 and 2 (Early stage OSCC)	NA	28 (37.8%)	44 (34.6%)
Stage 3 and 4 (Advanced stage OSCC)	NA	46 (62.2%)	83 (65.4%)
*Pathological T classification*			
T1	NA	5 (6.8%)	27 (21.3%)
T2	NA	34 (45.9%)	42 (33.1%)
T3	NA	3 (4.1%)	4 (3.1%))
T4	NA	32 (43.2%)	54 (42.5%)
*Pathological cervical lymph node involvement*			
Node negative (N0)	NA	46 (62.2%)	75 (59.1%)
Node positive (N+)	NA	28 (37.8%)	52 (40.9%)
*Pathological grade*			
Hyperplasia	18 (81.8%)	NA	NA
Mild dysplasia	2 (9.1%)	NA	NA
Moderate dysplasia	2 (9.1%)	NA	NA
Well	NA	7 (9.5%)	10 (7.9%)
Moderate	NA	47 (63.5%)	97 (76.4%)
Poor	NA	20 (27%)	20 (15.7%)
*Habit profile*			
Exclusive tobacco users	7 (31.8%)	51 (68.9%)	69 (54.3%)
Exclusive smoker	3 (13.6%)	1 (1.4%)	NA
Exclusive alcohol drinker	NA	NA	1 (0.8%)
Mixed habit**	8 (36.4%)	14 (18.9%)	34 (26.8%)
No Habit	NA	NA	3 (2.4%)
No information	4 (18.2%)	8 (10.8%)	20 (15.7%)

All the samples belongs to gingivobuccal complex (GBC) of oral cavity

T, Tumor classification based on size; N, Tumor classification based on lymph node metastasis

*IQR, Inter qaurtile range

**Mixed Habit: Tobacco chewing along with bidi/cigarette smoking and/or alcohol users

### Genome-wide DNA methylation analysis

We performed genome-wide DNA methylation in 22 normal buccal mucosa tissue samples from unrelated healthy subjects, 22 tissue samples from leukoplakia patients, and 74 tissue samples from gingivobuccal complex cancer patients using 850 K methylation arrays. Principal components analysis (PCA) of methylation sites in promoter regions identified two distinct clusters consisting of GBC-OSCCs and normals, respectively, while leukoplakia cases were spread across these two groups implying the absence of strong batch effects (Additional file 2: Figure S1).

Differential methylation analyses performed across CpG sites, CpG islands, promoters, and genes is summarized in Additional file 1: Table S4. The list of the top 20 hypomethylated and hypermethylated targets for CpG sites and islands based on combined ranks are presented in Additional file 1: Tables S5–S8. Top 20 hypomethylated and hypermethylated protein-coding genes and promoters based on combined ranks are shown in Additional file 1: Tables S9–S12.. In the site-level analysis, 2296 and 17,076 differentially methylated CpG sites were reported in leukoplakia and GBC-OSCCs, respectively. Of these, 1885 (82.10%) were hypermethylated, and 411 (17.90%) were hypomethylated in leukoplakia. Consistent with these observations, GBC-OSCCs also showed more hypermethylation (72.92%) than hypomethylation (27.08%). By genomic location, the proportion of differential methylation was highest for chromosome 19 in leukoplakia (10.80%) and chromosome 1 in GBC-OSCCs (8.47%), whereas chromosome 21 showed the lowest differential methylation in both (0.78% and 0.67%, respectively; Additional file 2: Figure S2).

Promoter hypermethylation is one of the major mechanisms of gene regulation; hence, our analysis focused on methylation in promoter regions. Differentially methylated promoters (DMPs) are illustrated using volcano plots. Additional file 2: Figure S3 depicts the increase in aberrant methylation as the lesion progresses from OPMD to OSCC. We did not observe any significant associations of DMPs with clinicopathological parameters such as tumour stage, nodal status, and recurrence. Similar to CpG sites, the highest number of DMPs were located on chromosome 19 in leukoplakia (10.28%) and on chromosome 2 in GBC-OSCCs (7.53%), with chromosome 21 having the lowest DMPs in both conditions (Additional file 2: Figure S4). Further, we identified 846 DMPs (543 hypermethylated, 303 hypomethylated) in leukoplakia and 5111 DMPs (1984 hypermethylated, 3127 hypomethylated) in tumors.

The genes associated with the DMPs were enriched for their involvement in several biological processes. Both leukoplakia and GBC-OSCC shared hypermethylated promoters involved in the regulation of transcription, gene expression, biosynthetic and metabolic processes. The hypomethylated promoters were associated with the development, cell adhesion, and regulation of immune-mediated responses (Additional file 1: Tables S13–S14).

### DNA methylation changes in leukoplakia and gingivobuccal complex cancers

**Fig. 1:**
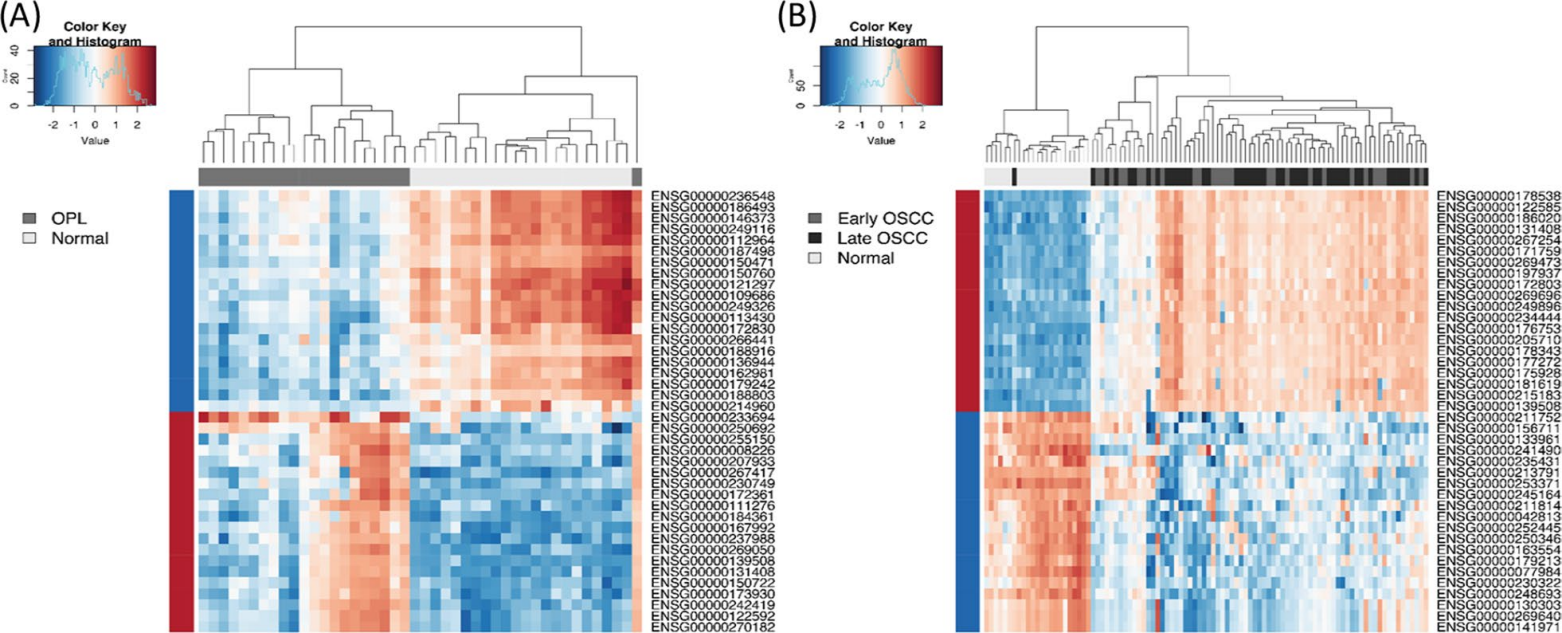
Heatmap of top 20 hypo- and hypermethylated promoters in leukoplakia and OSCC. **A** Differentially methylated promoters in OPL (grey) when compared to normal (light grey). **B** Differentially methylated promoters in early-stage OSCC (grey) and advanced-stage OSCC (dark grey) when compared to normal (light grey). The hypo- and hypermethylated promoters are represented in blue and red, respectively. All methylation values were log-transformed and scaled

The Venn diagram in Additional file 2: Figure S5 (A) depicts DMPs identified by comparison between (1) OPL vs normal, (2) tumor vs normal, and (3) tumor vs OPL. The heatmap of the top 20 hyper and hypomethylated promoters in leukoplakia and OSCC revealed a methylation signature different from normal, including promoters of well-established cancer genes such as *PPP1R1C, EID3, DLEC1, HOXA7, CDKN1B, MIR9-1, TSHZ3, FAM84A* and *CDH4* in leukoplakia. Similarly, hypermethylation of *SHISA3, KCNA3, NPY,* and hypomethylation of *NUMB, BST2, MAPK13* promoters, which have previously been implicated in other cancers, robustly separate normal and GBC-OSCC samples (Fig. 1). Selecting the top 200 DMPs and retaining only protein-coding genes in common between leukoplakia and GBC-OSCC revealed 45 hypermethylated promoters (Additional file 1: Table S15). Hypermethylation of TSGs, such as *CDKN1B, NR1H2, ZFP82,* and *SHISA3,* and other novel gene promoters might play important roles during oral carcinogenesis. These shared 45 promoters were also differentially methylated in a separate comparison between oral potentially malignant disorders and early-stage GBC-OSCC suggesting that these are early methylation events and may facilitate early detection of potentially cancerous lesions (Additional file 2: Figure S5(B)). We did not detect any significant DMPs between early- and late-stage GBC-OSCC. Reassuringly, we found substantial overlap between our study and previously published studies (Additional file 1: Table S1) in which established promoter methylation markers such as *FGF3, SOX17, RUNX1, WT1* were found to be associated with clinicopathological features.

### Integrative analysis

To find prognostic markers of GBC-OSCC, we performed an integrative analysis of DNA methylation with GE and CNA [6, 7] data using the R-package CNAmet. CNAmet facilitates the identification of putative driver genes, which are synergistically regulated by methylation and CNA. The integrative analysis by CNAmet provided gene lists in four categories summarized in Additional file 1: Table S16. We found 17 genes with hypomethylation or copy number gain and increased expression, including cancer-associated genes such as *AGO1, GHR* and *FAT1,* which might be potential oncogenes. Similarly, we identified 57 potential TSGs, of which at least 13 have been implicated in cancer, including *HOXB13, ZNF350, ZNF331, CNTNAP2, NEFH,* and *ABR* with reduced expression due to hypermethylation or loss. In addition, we found 21 genes with hypermethylation or gain and three genes with hypomethylation or loss, which are less common and are usually indicative of a methylation change farther from the promoter. These 21 genes with hypermethylation or gain included oncogenes *UCHL1, MLLT11, SLC35F2, SEMA6D,* and *ARHGEF39* and tumor suppressors *SOD2, RASSF2*, and *TRIM13*. The hypomethylation or loss category included three genes, none of which are known drivers in cancer.

### Survival analysis

We applied regularised Cox regression with L1 penalty (LASSO) on the methylation and CNA data of the genes selected by CNAmet to identify potential survival-associated markers. We found 32 candidate prognostic markers (Table 2). The top predictors based on LASSO estimates included *FAT1, JAK3, ARHGEF39, CD1C, HOXB13, GLDC,* and *TRIM13*. For most genes in Table 2, exactly one of the methylation changes or the CNA is associated with a change in survival; the three genes for which both changes are associated with survival are *FEZ2*, *MLLT11*, and *ZNF350*. We validated 32 candidate prognostic markers from integrative analysis using the TCGA_HNSC set from the MethCNA database (Additional file 1: Table S17). We found 87.5% of genes were in agreement with methylation status from our data and TCGA cohort, except for *FAT1, GHR, LY75,* and *MED7.* Overall, we observed high concordance between our analysis of our data and the TCGA data strengthening our results.

**Table 2:** List of candidate prognostic markers based on methylation and CNA data

Sr no	Cytoband	Genes	Alteration	Promoter status	MethCNA annotation	∆β	Rnbeads adjusted FDR	CNAmet FDR	LASSO Coefficients
1	1p34.3	*AGO1*	Gain	Hypo	Loss tendency*	− 0.13040191	9.92E−08	0	0.02357709
2	9p13.3	*ARHGEF39*	Hyper	Hyper	Not available	0.15826579	4.79E−11	0.07915789	5.406079817
3	1q23.1	*CD1C*	Hypo	Hypo	Not available	− 0.10967453	0.00000036	0.025	− 5.86758818
4	7q21.13	*CLDN12*	Gain	Hypo	Gain	− 0.20024732	3.01E−10	0.07425	− 0.17467983
5	20p11.21	***CST7***	Gain	Hypo	Gain	− 0.18512047	3.09E−17	0.07826087	0.7816124
6	18q22.3	***CYB5A***	Loss	Hyper	Loss	0.154479225	0.00000204	0	− 0.2566186
7	9q34.11	*DPM2*	Gain	Hyper	Gain	0.110067901	3.95E−09	0.01342857	0.020726005
8	10p14	*ECHDC3*	Loss	Hyper	Loss	0.102629326	0.000083	0.051625	0.2538569
9	4q35.2	*FAT1*^*#*^	Hypo	Hypo	Hyper	− 0.10716469	2.15E−09	0	− 8.91100971
10	2p22.2	*FEZ2*	Gain	Hypo	No effect	− 0.12593147	0.00000482	0.07772727	− 0.09276379
11	2p22.2	*FEZ2*	Hypo	Hypo	No effect	− 0.12593147	0.00000482	0.07772727	− 0.42588184
12	5p13.1-p12	***GHR***	Gain	Hypo	Gain	− 0.12027275	9.11E−13	0	0.49366068
13	9p24.1	*GLDC*^*#*^	Hyper	Hyper	Hyper	0.11272417	0.000540999	0.02145455	1.043842
14	17q21.32	*HOXB13*^*#*^	Hyper	Hyper	Hyper	0.101298897	0.000789507	0	3.113007
15	19p13.11	*JAK3*	Hyper	Hyper	Hyper	0.106127081	0.0000126	0.06807692	8.206031
16	2p23.3	*KRTCAP3*	Hyper	Hyper	Hyper	0.103330448	0.00000192	0.00680769	1.10005
17	1p34.1	*LURAP1*	Loss	Hyper	Not available	0.115584283	0.00000258	0	0.03147996
18	2q24.2	***LY75***	Loss	Hyper	Loss tendency*	0.154711559	0.00000465	0	− 0.3404406
19	9q34.3	*MAMDC4*	Hyper	Hyper	Not available	0.103718753	1.66E−12	0	0.9015477
20	5q33.3	*MED7*	Hypo	Hypo	Hyper	− 0.10052829	0.000350364	0.047	− 0.22621307
21	1q21.3	*MLLT11*	Hyper	Hyper	Hyper	0.1010586	0.0000537	0.06042857	0.331861046
22	1q21.3	***MLLT11***	Gain	Hyper	Gain	0.1010586	0.0000537	0.06042857	0.599877098
23	6q23.2	*TAAR5*	Hypo	Hypo	Not available	− 0.10711508	0.00000875	0.06266667	− 5.26569245
24	2q24.2	*TANK*	Gain	Hyper	Loss tendency*	0.127622181	0.00000758	0	− 0.42285359
25	13q14.2	*TRIM13*	Hyper	Hyper	Hyper	0.104869077	0.000000876	0.08952381	1.044432765
26	3q23	*U2SURP*	Gain	Hyper	Not available	0.178676248	9.68E−11	0	− 0.16037072
27	19q13.41	*ZNF83*	Loss	Hyper	Gain tendency*	0.165017242	3.09E−11	0.04895745	2.93189E−16
28	19q13.41-q13.42	*ZNF160*	Loss	Hyper	Gain tendency*	0.284229499	1.28E−15	0.06726	0.0111227
29	19q13.42	*ZNF331*	Loss	Hyper	Gain tendency*	0.218690004	2.06E−15	0.0122069	6.15697E−15
30	19q13.41	*ZNF350*	Loss	Hyper	Gain tendency*	0.137293195	0.000000187	0.0122069	0.4037165
31	19q13.41	*ZNF350*	Hyper	Hyper	Hyper	0.137293195	0.000000187	0.0122069	− 2.244252
32	7p22.1	*ZNF853*	Gain	Hyper	Gain	0.156897804	4.03E−10	0.0282	0.025609823

The predictors (gene promoter methylation patterns and gene copy number alteration) are outlined by the gene symbol and alteration columns. The MethCNA annotation column summarises the alterations observed in TCGA head and neck squamous cell carcinoma (HNSCC) according to the MethCNA database

*The difference between the observed frequencies of both loss and gain is small in the MethCNA database for these genes

^#^Target gene promoter selected for validation using bisulfite pyrosequencing; genes represented in bold were selected for copy number analysis using TaqMan real time PCR

### Validation of candidate marker genes

**Fig. 2:**
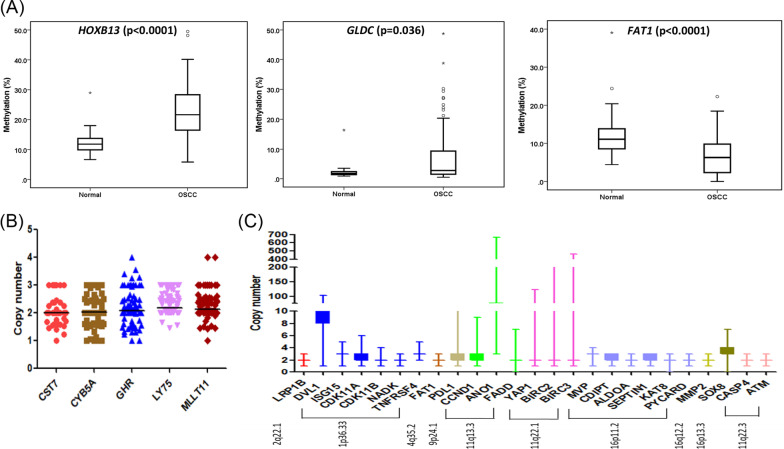
Validation of promoter methylation and copy number targets. **A** Pyrosequencing analysis showing differential promoter methylation between normals and OSCC for *HOXB13*, *GLDC,* and *FAT1*. **B**, **C** panel demonstrates the frequency of copy number targets selected from integrative analysis and previously published reports^6,7^

To validate the results of the integrative analysis, we performed bisulfite pyrosequencing for promoter methylation and TaqMan real-time PCR for CNAs. Three genes were selected based on their role in other cancers, namely *HOXB13, GLDC,* and *FAT1*. Pyrosequencing of their gene promoters was performed in independent samples of 127 GBC-OSCCs and 20 healthy controls. The CpG site of interest covered by each sequencing primer is listed in Additional file 1: Table S18. Figure 2A shows the average percentage methylation values for each of the promoters compared to normals. Significant promoter hypermethylation was observed in *HOXB13* (*P* < 0.0001), *GLDC* (*P* = 0.036), and hypomethylation was observed in *FAT1* (*P* < 0.0001). Thus, the pyrosequencing data confirmed the findings based on the methylation array data. There was no significant difference in methylation levels between early and late-stage OSCC except for *FAT1* (Additional file 2: Figure S6).

For validation of CNAs, we performed real-time PCR for five candidate genes from the integrative analysis along with additional genes from previously reported chromosomal locus associated with poor survival [6, 7]. The detailed CNA frequency of each target compared to control DNA and CNAs of the TCGA-HNSCC dataset from the Genome Data Commons (GDC) Data portal are represented in Additional file 1: Table S19 and Fig. 2B, C. The majority of the samples did not show a CNA for the targets from the integrative analysis. The frequencies of copy number gains were 3.1–11% of samples for *CST7, GHR,* and *MLLT11*; in addition, 5.5% of samples showed copy number losses for *CYB5A*. Interestingly, *LY75* showed copy number gains (17.3%) instead of the expected losses. The gain/amplification was highest for *DVL1* (95.9%) and *ANO1* (100%) from the 1p36.32 and 11q13.3 loci, respectively. The genes on the 11q22 amplicon, *BIRC2, BIRC3,* and *YAP1,* also showed high copy number gains and amplification in some samples.

We analysed the association between validation targets and clinicopathological parameters Additional file 1: Table S20. We found that *NADK* gain (BH *P-*value = 0.044) was associated with the pathological stage. Further, we studied the correlation of biomarkers among themselves Additional file 1: Table S21. We found *ISG15* and *NADK* (R = 0.665) on band 1p36.33 showed moderate correlation. A strong correlation was observed between *BIRC2* and *BIRC3* (R = 0.778) as well as *BIRC2* and *YAP1* (R = 0.884) present in the 11q22 locus.

**Fig. 3:**
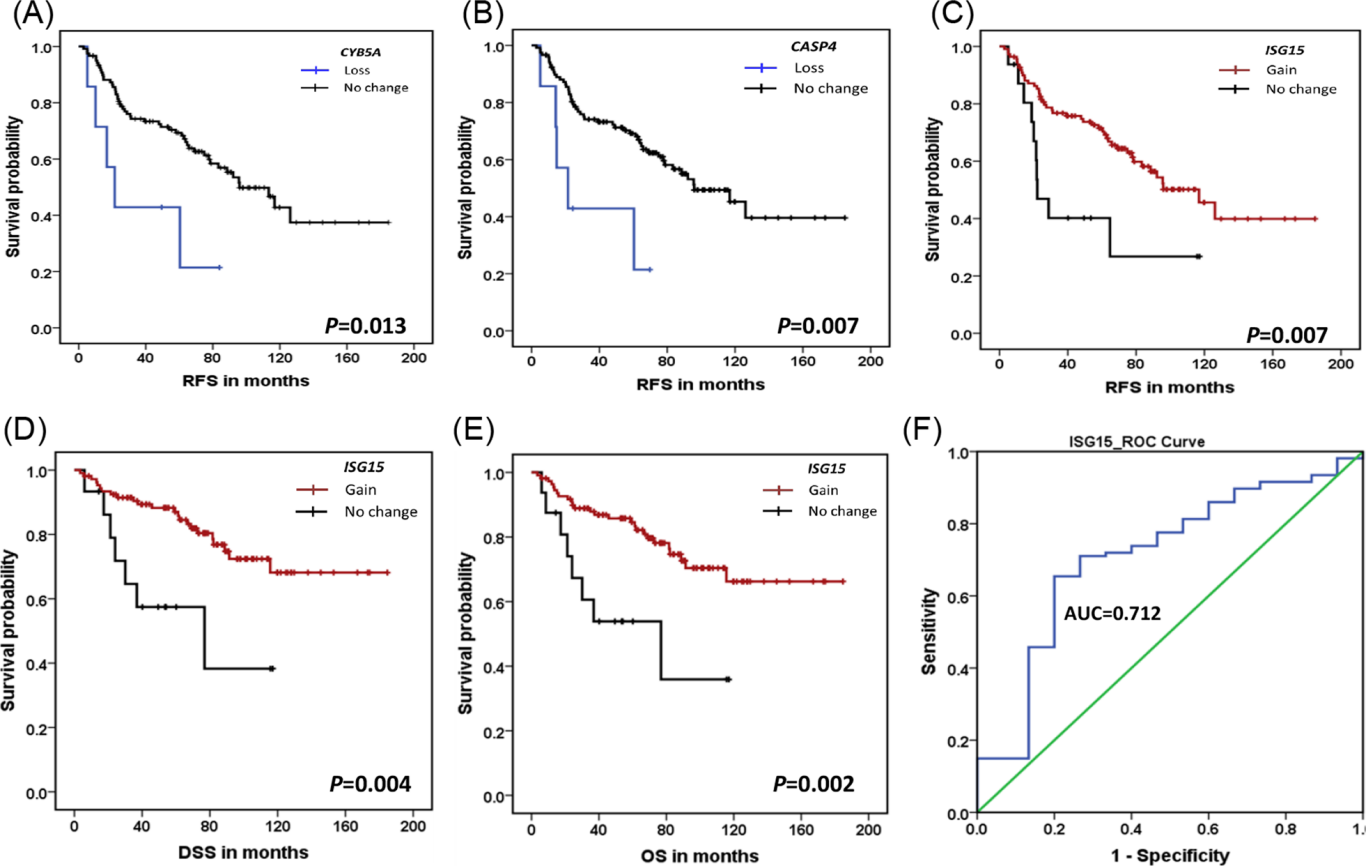
Kaplan–Meier (KM) plots and receiver operating characteristic (ROC) curve. KM plots for **A** *CYB5A* and **B** *CASP4* losses were associated with poor recurrence free survival (RFS). *ISG15* gain showed better **C** RFS, **D** Disease specific survival (DSS) and **E** Overall survival (OS). **F** illustrates the prognostic potential of *ISG15* as analysed by a ROC curve

To evaluate the association of biomarkers with clinical outcomes, Kaplan–Meier (KM) survival analysis was performed using the log-rank test. KM plots are provided in Fig. 3A–E. We did not observe a strong correlation between methylation and CNA targets selected from the integrative analysis to clinical outcomes. However, we observed a trend toward poor survival. Univariate Cox regression analysis (Additional file 1: Table S22) performed on validation targets indicated that *ISG15* may be associated with better clinical outcomes. A gain in *ISG15* was nominally significantly associated with better recurrence-free survival (RFS) [HR (95% CI):0.40 (0.2–0.80), *P* = 0.010], disease-specific survival (DSS) [HR:0.30 (0.13–0.71), *P* = 0.006] and overall survival (OS) [HR:0.31 (0.14–0.69), *P* = 0.004], but not after correction for multiple testing. In addition, *CYB5A* [HR:3.067 (1.21–7.76), *P* = 0.018] and *CASP4* [HR:3.35(1.32–8.55), *P* = 0.011] were nominally associated with poor RFS. Multivariable analysis with nominal *P*-values adjusted for the confounding factors revealed *ISG15* was associated with RFS [HR:0.47 (0.23–0.99), *P* = 0.049], DSS [HR:0.32 (0.13–0.77), *P* = 0.011] and OS [HR:0.33 (0.15–0.75), *P* = 0.008], Table 3. The predictive value of *ISG15* was determined by the receiver operator characteristic (ROC), which showed an area under the curve (AUC) of 0.711 (95% CI 0.57–0.85, *P* = 0.007), 0.712 (95% CI:0.57–0.85, *P* = 0.008) and 0.715 (95% CI 0.58–0.85, *P* = 0.006) for RFS, DSS, and OS, respectively. The optimal cutoff value determined using Youden’s index for DSS was 60.67 with a sensitivity of 65.4% and a specificity of 80%, Fig. 3F. Further, we stratified the validation data according to node and stage to assess the robustness of prognostic markers. Stratification according to nodal status and stage revealed new prognostic markers Additional file 1: Table S23–S25. Univariate Cox regression in N0 samples showed that *BIRC2, BIRC3,* along with other markers, were associated with poor RFS, DSS, and OS, suggesting different molecular signatures of N0 and N + tumors.

**Table 3:** Prognostic significance of biomarkers

Variables	Univariate Cox analysis		Multivariable Cox analysis	
	HR (95% CI)	*P* value	HR (95% CI)	*P* value
*Recurrence free survival (RFS)*				
Age (below 60 vs 60 & above)	1.73 (0.97–3.01)	0.062	2.32 (1.24–4.34)	0.008
Sex (male vs female)	0.74 (0.33–1.64)	0.456	0.52 (0.22–1.22)	0.131
Stage (early vs advanced)	1.46 (0.83–2.56)	0.187	1.11 (0.52–2.37)	0.781
Lymph node (negative vs positive)	1.43 (0.85–2.40)	0.181	1.36 (0.66–2.81)	0.400
*CYB5A* (no change vs loss)	3.067 (1.21–7.76)	0.018	2.47 (0.82–7.44)	0.108
*CASP4* (no change vs loss)	3.35 (1.32–8.55)	0.011	1.92 (0.63–5.80)	0.250
*ISG15* (no change vs gain)	0.40 (0.2–0.80)	0.010	0.47 (0.23–0.99)	**0.049**
*Disease specific survival (DSS)*				
Age (below 60 vs 60 & above)	1.62 (0.74–3.54)	0.229	1.76 (0.74–4.17)	0.202
Sex (male vs female)	1.30 (0.53–3.18)	0.567	0.91 (0.34–2.47)	0.854
Stage (early vs advanced)	1.64 (0.75–3.60)	0.214	1.14 (0.40–3.29)	0.794
Lymph node (negative vs positive)	1.65 (0.80–3.39)	0.169	1.44 (0.54–3.84)	0.463
*ISG15* (no change vs gain)	0.30 (0.13–0.71)	0.006	0.32 (0.13–0.77)	**0.011**
*Overall survival (OS)*				
Age (below 60 vs 60 & above)	1.56 (0.74–3.27)	0.240	1.58 (0.70–3.55)	0.269
Sex (male vs female)	1.37 (0.59–3.14)	0.464	0.99 (0.40–2.51)	0.993
Stage (early vs advanced)	1.90 (0.89–4.09)	0.100	1.49 (0.57–3.94)	0.418
Lymph node (negative vs positive)	1.63 (0.83–3.19)	0.156	1.22 (0.51–2.91)	0.652
*ISG15* (no change vs gain)	0.31 (0.14–0.69)	0.004	0.33 (0.15–0.75)	**0.008**

The values in bold are to indicate that these are highly significant changes where a p-value less than 0.05 isconsidered statically significant

## Discussion

We have presented a comprehensive study that provides genome-wide methylation profiles associated with oral premalignant lesions and gingivobuccal complex cancers. We report a methylation signature of leukoplakia and GBC-OSCC, which can be used for identifying high-risk precancerous lesions having the potential of malignant transformation. Another feature of the study is that we integrated methylation data with genomic copy number data and transcriptomic data to identify 32 genes with prognostic significance synergistically regulated by copy number and methylation. We report previously unrecognized differential methylation of *FAT1*, *HOXB13,* and *GLDC* in GBC-OSCC tissues. Further, validation experiments revealed that losses of *CYB5A* and *CASP4* were associated with poor RFS, while gain of *ISG15* was identified as a potential prognostic marker for better RFS, DSS, and OS.

We performed genome-wide DNA methylation analysis that reported 846 (303 hypomethylated, 543 hypermethylated) and 5111 (3127 hypomethylated, 1984 hypermethylated) DMPs in leukoplakia and GBC-OSCCs, respectively. The methylation profile of leukoplakia and tumors are different from normals, and methylation aberrations increase as the lesion progresses. The top 200 DMPs common between leukoplakia and tumor and retaining only protein-coding genes revealed 45 hypermethylated promoters. In our literature search, we found consistent previous reports regarding 21 of these 45 genes (Additional file 1: Table S15). Among the genes adjacent to these promoters, *CDKN1B, ZFP82, SHISA3, GPX7,* and *IRF8* are known TSGs [21–25]. The hypermethylation of *KCNA3*, *IRF8,* and *ZNF529* was also reported by Foy et al. [26] in leukoplakia transforming into OSCC, implying important early events which might play a role in disease progression. Hence, these markers can be helpful in stratifying potentially malignant lesions and need to be further investigated. Nevertheless, there is a significant overlap between our findings and previously published reports on methylation in OSCC (Additional file 1: Table S1); many of these differentially methylated genes were reported in an extensive review by Flausino et al. [27].

The transformation of leukoplakia to malignant carcinoma involves the accumulation of genomic and epigenomic alterations [28–32]. The recurrent CNA (gains and losses) and aberrant methylation (hypo- and hypermethylation) have been linked to apoptosis evasion, metastasis, and therapy resistance [33, 34]. Hence, to search for prognostic biomarkers, we integrated DNA methylation, GE, and CNA and identified 32 candidate marker genes with potential prognostic value in gingivobuccal complex cancers. Hypomethylation and amplification-dependent gene upregulation were observed for seven potential oncogenes associated with poor survival. Of these seven genes, hypomethylation of *FAT1* and *GHR* was also observed in leukoplakia, indicating early oncogenic events. *FAT1* regulates cell–cell contact and acts as a tumor suppressor or oncogene in a context-dependent manner [35]. Multiple studies reported mutation, deletion, and hypermethylation of *FAT1* in OSCC [36–39]. Conversely, upregulation has been linked with cell migration and invasion through β-catenin localisation in oral cancers [40]. A recent study in OSCC reported that *FAT1* may act as an oncogene, and its overexpression was associated with poor prognosis affecting proliferation, migration, and apoptosis [41]. Here, for the first time, we report that *FAT1* is regulated by promoter hypomethylation that could lead to its overexpression, supporting its oncogenic role in OSCC. The growth hormone receptor (*GHR)* is overexpressed in many solid tumors [42–44] and promotes tumor proliferation, progression, and metastasis in breast cancer via the BRAF/MEK/ERK pathway [44]. We observed a *GHR* copy number gain in 10% of OSCC tissues. It has been suggested that the knockdown of *FAT1* and *GHR* enhances the sensitivity toward drugs, making them therapeutic drug targets [45–47]. Other genes from this category are *CST7, CD1C, AGO1, CLDN12,* and *FEZ2*. *CST7* and *CD1C* are involved in immune modulation [48, 49]. Hypomethylation or gain of *CST7* has not been reported in OSCC but has been proposed as a prognostic biomarker for other carcinomas [50]. Previously, upregulation of *AGO1* and *CLDN12* has been linked to progression and metastasis [51, 52]. Both hypomethylation and gain of *FEZ2* were associated with poor survival, consistent with a report by Yang et al. [53] in pancreatic ductal adenocarcinoma (PDAC). Overall, the CNA and methylation status of these genes can serve as a guide for the identification and design of therapeutic drug targets.

In the hyperloss category, we identified 13 potential TSGs linked with poor clinical outcome. We found 4 genes (*LY75, ZNF83, ZNF160, ZNF331*) common in leukoplakia and OSCC, while 9 genes appeared only in OSCC, suggesting their role in disease advancement. Among these, *HOXB13* (involved in skin development) and *GLDC* (a component of the glycine cleavage system) are both known TSGs that are epigenetically silenced in many cancers [54–56] and promote apoptosis and autophagy [57, 58], respectively. However, no literature evidence is available for the methylation status of these genes in the context of oral cancer. We found *HOXB13* and *GLDC* to be novel TSGs in OSCC, inactivated through promoter hypermethylation. Further, we validated CNAs of *CYB5A* and *LY75*. *CYB5A* (18q22.3) is a membrane-bound mitochondrial hemoprotein involved in regulating cellular redox balance [59]. The significant downregulation of *CYB5A* has been reported in cancers [60, 61]; however, the mechanism underlying the antitumor effect of *CYB5A* has not been explored much. *CYB5A*is reported to be deleted, induces autophagy-mediated cell death in PDAC [62]. Consistent with this report in PDAC, we found loss of *CYB5A* to be associated with shortened patient survival. *LY75* encodes a mannose receptor involved in the immune and inflammatory response [63]. Recently, Mehdi et al. [64] showed hypomethylation and overexpression of *LY75* regulate EMT phenotype and metastatic potential in ovarian cancer. Interestingly, we observed copy number gains in *LY75* rather than losses. Another significant gene in this category, *JAK3,* is involved in the JAK-STAT signalling pathway, reported to be methylated in bladder cancer [65]. Lastly, we also reported a cluster of zinc finger proteins *(ZNF350, ZNF331, ZNF160, ZNF83)* located on 19q13.41, which belong to a Krüppel-type family of transcriptional repressors, and have previously been shown to be epigenetically silenced and downregulated in multiple cancers [66, 67] including head and neck cancers [68]. The remaining genes in the hyperloss category are *ECHDC3, KRTCAP3, LURAP1,* and *MAMDC4,* none of which are known drivers in cancer.

In the hypergain category, we identified seven genes. Interesting candidates include *MLLT11* and *TRIM13,* whose CNAs or differential methylation status have not been reported in OSCC*. MLLT11* (1q21.3) is an oncogene dysregulated in some acute myeloid leukemia patients with t(1;11)(q21;q23) translocation and also facilitates the progression of solid tumors [69, 70]. Recurrent gains of the 1q21.3 region and upregulation of *MLLT11* were reported in the Wilms tumor [71]. We report *MLLT11* CNA in 11% of OSCC samples. *TRIM13* is a member of the tripartite motif (TRIM) family downregulated in multiple neoplasms [72–74] and acts as TSG by inducing apoptosis [73]. *MLLT11* has been shown to play a dual function in malignancy, including both promotion and inhibition of cancer progression, and therefore may be relevant in OSCC prognosis and management. While *MLLT11* acts as an oncogene, *TRIM13* acts as a TSG; hence genes from the hypergain category are enigmatic and can be interpreted only with the aid of additional functional studies. Lastly, in the hypoloss category, we did not find any literature evidence for *MED7* and *TAAR5*.

Numerous studies have identified prognostic factors such as tumor size, cancer stage, surgical margins, nodal involvement, grade, perineural invasion, lymphovascular invasion, and extranodal extension associated with disease-free survival and overall survival [75–79]. Previously, recurrent focal alterations in OSCC were found to be associated with poor survival; however, the prognostic significance of CNAs at the single-gene level remains to be elucidated [6, 7]. Hence, in search of novel biomarkers, we validated important targets from previous studies and observed that CNA status differs for each gene even though the nearby genes are present on the same amplicon. In this study, copy number loss in *CASP4*, and gain in *ISG15* were associated with survival. Amplification of the 1p36.33 locus was found in progressive leukoplakia [30] as well as in OSCC [7]. Particularly, Interferon Stimulated Gene 15 (*ISG15*) present on 1p36.33 locus encodes a ubiquitin-like protein associated with antiviral response and regulation of key cellular processes [80] and has prognostic significance in multiple cancers [81–83]. The ubiquitin domains in ISG15 make it possible for ISG15 to be conjugated to some other proteins via lysine residues. The conjugated ISG15 (ISGylation) has pro-tumorigenic activity, while the extracellular free form has anti-tumorigenic activity in breast cancer [84] and is being considered as a tumor-associated antigen for cancer immunotherapy [85]. In OSCC, *ISG15* was reported to be upregulated and to affect migration and lymph node metastasis [86]. Interestingly, for the first time, we report that a gain of *ISG15* is associated with better relapse-free, disease-specific, and overall survival, making it a potential prognostic marker of OSCC suitable for further validation. In our study, loss of *CASP4* and the tumor suppressor *ATM* on 11q22.3 was associated with lymph node involvement. *CASP4* is a component of non-canonical inflammasome that induces pyroptosis [87]. Previous studies have demonstrated that higher expression of *CASP4* can serve as a prognostic marker in glioma and renal cell carcinoma [88, 89]. Alternatively, few studies have reported the loss and downregulation of *CASP4* was associated with poor prognosis in head and neck squamous cell carcinoma [90–92]. Similar to these findings, we also observed the loss of *CASP4* was associated with poor RFS.

Although the data presented here indicate the interplay between CNA, GE, and methylation, there are some limitations. The CNAmet software may have missed genes that are not regulated synergistically, co-expressed, or co-methylated. This study underlines the need for functional validation of such datasets in an independent cohort. The clinical outcome of leukoplakia patients was not available; hence they were not included for further validation. Furthermore, while performing genome-wide methylation analysis, it is important to note that the differentially methylated sites and regions reported are sensitive to the choice of |∆β| threshold (Additional file 1: Table S26). Given the reduced spread of $\Delta \beta$ in DMRs in our study, we use a more relaxed threshold to be more inclusive for the downstream integrative analysis. However, exploring a less permissive threshold and shifting |∆β| threshold from 0.2 to 0.25 for genome-wide CpG sites, we drop two-thirds of the hypermethylated sites. Similarly, moving the |∆β| threshold from 0.1 to 0.25, we retain only 2 and 62 DMPs in leukoplakia and GBC-OSCCs, respectively. While we recover several top established DMPs, including, *KCNA3, NPY, SHISA3, ZNF529* from the literature, and *ZNF160* from the integrative analysis, a stricter threshold increases the chance of dropping several well-studied and prospective markers. This is exemplified by further moving the |∆β| to 0.5, which resulted in only 9 differentially methylated sites in GBC-OSCCs and no significant regions. Thus, there is a trade-off between sensitivity and specificity. A quantitative study exploring this trade-off could help improve the discovery of prospective markers.

Despite these concerns, the strength of the study relies on the integration of three data types from the same patients, enabling a more comprehensive understanding of the underlying molecular mechanisms in oral carcinogenesis [93]. A key strength of our study design is that we have utilized normal samples from healthy individuals rather than noncancerous tissue adjacent to cancerous tissue from affected individuals to avoid the effects of field cancerization [94]. Another strength is that we used only tumors from GBC sites to avoid heterogeneity since different anatomic sites have different methylation profiles [95]. Lastly, in contrast to many previous studies, our study also validated selected candidates in an independent set of samples, further bolstering our findings.

## Conclusion

Overall, our study identified potential candidate genes in oral carcinogenesis, which needs functional validation. The global methylation signature common between leukoplakia and gingivobuccal complex cancers may help in identifying potential malignant lesions. Differential promoter methylation analysis revealed *FAT1, GLDC*, and *HOXB13* to be associated with oral carcinogenesis. Copy number changes in *CYB5A*, *CASP4*, and *ISG15* were found to be potential predictors of survival. The data provides novel insight into OSCC carcinogenesis and prognosis. This study highlights the importance of integration of data across the genomic, epigenomic, and transcriptomic levels to identify novel genes with prognostic and therapeutic potential in gingivobuccal complex cancer.

## Materials and methods

### Sample collection and micro-dissection

The institutional ethics committee of Tata Memorial Hospital approved the study (Project No-218 of 2016). Written informed consent was obtained from all study participants. Frozen tissues of treatment-naïve, pathologically diagnosed, and surgically resected gingivobuccal tumor and leukoplakia tissues were obtained from Tata Memorial Hospital. The non-inflamed gingivobuccal tissues obtained from healthy individuals with no previous history of cancer were obtained from Nair Dental College. Cryosectioning, DNA-RNA extraction and HPV detection were performed as described in the Additional file 3: Additional informations. The training set included 74 OSCCs, 22 leukoplakia, and 22 normals, and the validation set consisted of 127 GBC-OSCCs and 20 normals.

### DNA methylation profiling and analysis

Genome-wide DNA methylation profiling was performed on 74 GBC-OSCC samples, 22 leukoplakia samples, and 22 normal tissues from healthy individuals using Infinium Methylation EPIC (850 K) BeadChips (Illumina, San Diego, CA, USA). The bisulfite conversion of gDNA and hybridization details are provided in the Additional file 3: Additional informations. The raw files have been submitted to the Gene Expression Omnibus (GEO) with accession number GSE204943.

DNA methylation data (.IDAT files) were processed and analysed using the R Bioconductor packages RnBeads (v 1.12.1) and RnBeads.hg19 (v 1.13.1). The RnBeads pipeline included quality control assessment, preprocessing, normalization, exploratory, and differential methylation analyses. Through quality control, preprocessing, and normalization steps, after filtering SNP-enriched probes, probes with unreliable measurements (Greedycut algorithm), context-specific probes, and other filtering procedures, 820,193 probes were retained across 118 samples. Differential methylation analyses were performed across CpG sites, CpG islands, promoters, and genes. The standard methylation *β* values are defined as the ratio of methylated to overall probe intensities and summarize the methylation status of each probe. RnBeads also combines data for multiple probes in a CpG island, in a promoter, or in a gene so that each of these types of genomic units gets a single averaged *β* value in each sample. Combined ranks were calculated based on differences in mean methylation, log ratio in mean methylation, and *P*-value from limma or t-test. Genome-wide CpG sites, promoters, CpG islands, and genes with adjusted false discovery rate (FDR) *P*-values < 0.05 and |∆*β*|> {0.2, 0.1, 0.1, 0.1}, respectively, were reported as significantly differentially methylated. The RnBeads pipeline also performed gene set enrichment analyses.

### Integrative analysis

We used the R-package CNAmet [96] to integrate methylation data with previously published CNA (accession numbers-GSE85514, GSE23831) and GE data (accession numbers-GSE85195, GSE23558) [6, 7]. CNA calls were previously done with GISTIC [97]. Since CNAmet requires continuous gene expression data and binary copy number gain, copy number loss, hyper-, and hypomethylation data, all of the equal dimensions, the data were processed as follows. We focused on significant DMPs of protein-coding genes in OSCC and subsequently identified 449 common protein-coding genes with expression, methylation, and CNA data shared across 58 tumor samples. We prepared the four binarized matrices- hypomethylation, hypermethylation, loss, and gain as follows. The *β* values of the promoters in GBC-OSCC samples were first standardized with respect to the mean and standard deviation of normal samples (z-score). A promoter in a sample was considered to be hypomethylated and assigned a value of 1 if the z-score was less than − 2.576, and 0 otherwise. Similarly, a promoter in a sample was considered to be hypermethylated and assigned a value of 1 if the z-score was greater than 2.576, and 0 otherwise. The chosen cut-offs ensured the inclusion of the most differentially methylated promoters. For the CNA data, a gene in a sample was considered deleted and assigned a value of 1 if the GISTIC value was in {− 1, − 2}, and 0 otherwise. Similarly, a gene in a sample was considered amplified and assigned a value of 1 if the GISTIC value was in {1, 2}, and 0 otherwise. For GE, we used previously published data [6, 7]. Subsequently, we evaluated the effects of all four combinations of methylation and CNA data (hypermethylation with loss, hypomethylation with loss, hypermethylation with gain, and hypomethylation with gain) on gene expression using CNAmet with default parameters. For each combination of methylation and CNA data, CNAmet prioritizes the genes (CNAmet hits) according to a synergistic score, and a corresponding FDR corrected *P*-value. Literature-supported evidence was obtained by querying CNAmet results on CancerMine [98].

### Survival analysis

We retained the top significant genes (FDR corrected *P*-value < 0.1) that were in agreement with the methylation status from our differential methylation analysis and performed survival analysis on these hits. We used disease-specific survival times recorded in months for 17 dead of disease (DOD events) samples and 53 right-censored tumor samples. For each combination of CNAmet hits, we created a design matrix with stage, sex, age, *ꞵ* values of the hits, and GISTIC values of the hits. We applied regularised Cox regression with L1 penalty (LASSO) to select prognostic markers associated with survival. We compared our data with the TCGA data for HNSC as represented in the MethCNA database [99]. MethCNA is a publicly available database that integrates genomic and epigenomic data from exactly the same DNA specimen. We particularly compared 32 candidate prognostic hits from integrative analysis to the promoters in head and neck squamous cell carcinoma (TCGA_HNSC). The TCGA_HNSC data includes cancer samples from all the anatomic sites of the head and neck region including the oral cavity, larynx, alveolar ridge, oropharynx, floor of mouth, hypopharynx, oral tongue, and tonsil.

### Bisulfite pyrosequencing

The promoter methylations of *HOXB13, GLDC,* and *FAT1* were validated by bisulfite pyrosequencing in an independent 127 GBC-OSCCs and 20 normal samples. In order to cover more CpG sites, we designed two primer sets for each gene targeting CpG locus represented by the 850 K BeadChip. The details of PCR amplification and bisulfite pyrosequencing are given in Additional file 3: Additional informations and Additional file 1: Table S18. The methylation percentages for each CpG locus in controls and GBC-OSCC cases were calculated by the PyroMark Q96ID Software (Qiagen).

### Copy number alteration (CNA) analysis for validation

The copy numbers of candidate genes were validated by TaqMan qPCR. The detailed protocol and selected copy number assays are provided in Additional file 3: Additional informations and Additional file 1: Table S27. We chose two copy number assays targeting different regions of the same gene to strengthen the results for targets from the integrative analysis. The copy number was calculated using CopyCaller software v2.1 (Applied Biosystems). The CopyCaller-predicted copy number was used for the analysis, and more than two copies were considered a gain. One copy was considered a partial loss, and zero copies was considered a complete loss.

### Statistical and survival analyses for validation

The statistical analysis of the validation set was performed using IBM SPSS v20 software. Mann–Whitney two-tailed tests were applied to compare methylation between tumor and normal. The categorical data are presented as frequency and percentage. We categorise the methylation data as hypermethylated (z-score ≥ 2.576) and hypomethylated (z-score ≤ − 2.576). The possibility of an association between different biomarkers and clinicopathological parameters was tested using the χ^2^ test and Fisher’s exact test wherever applicable. Correlation between pairs of biomarkers was computed using Spearman rank correlation. Survival was quantified as the number of days between surgery and disease-specific death (DSS), disease recurrence (RFS), or death due to any other cause (OS) and analysed by Kaplan–Meier curves and log-rank tests. The predictive value of *ISG15* gain associated with survival was determined by the area under the receiver operating characteristic (ROC) curve, and an optimal cutoff point was determined using the Youden index [100]. The univariate Cox proportional hazard models were fitted for all available variables separately, and a multivariable Cox model was then applied to adjust for the potential confounders age, sex, stage, and nodal status. All tests were performed two-tailed, and a *P*-value < 0.05 was considered nominally significant. All tests for association between targets and clinical outcomes, as well as for correlations among targets (Additional file 1: Tables S20–S24) were corrected for multiple testing using the Benjamini-Hochberg (BH) method to control the FDR at 0.05.

## Supplementary Information

**Additional file 1. Table S1**: Literature of genome wide DNA methylation analysis in OSCC. **Table-S2**: Detailed demographic and clinicopathological characteristics of the leukoplakia samples. **Table S3**: Detailed demographic clinicopathological and characteristics of the OSCC samples. **Table S4**: Summary of genomewide differentially methylated regions. **Table S5**: Top 20 differentially methylated CpG sites identified across the leukoplakia samples. **Table S6**: Top 20 differentially methylated CpG sites identified across the OSCC samples. **Table S7**: Top 20 differentially methylated CpG islands identified across the leukoplakia samples. **Table S8**: Top 20 differentially methylated CpG islands identified across the OSCC samples. **Table S9**: Top 20 differentially hypermethylated and hypomethylated promoters identified across the leukoplakia samples. **Table S10**: Top 20 differentially hypermethylated and hypomethylated promoters identified across the OSCC samples. **Table S11**: Top 20 differentially hypermethylated and hypomethylated genes identified across the leukoplakia samples. **Table S12**: Top 20 differentially hypermethylated and hypomethylated genes identified across the OSCC samples. **Table S13**: Gene enrichment analysis of top 100 differentially methylated promoters in leukoplakia based on combined rank. **Table S14**: Gene enrichment analysis of top 100 differentially methylated promoters in OSCC based on combined rank. **Table S15**: List of differentially methylated promoters common between leukoplakia and OSCC. **Table S16**: Candidate target gene list obtained from integrative analysis of copy number, gene expression, and DNA methylation data using CNAmet. **Table S17**: Comparison of our data with TCGA_HNSC cohort. **Table S18**: Details for pyrosequencing primers. **Table S19**: Copy number alteration distribution among candidate genes. **Table S20**: Association between biomarkers and clinicopathological parameters. **Table S21**: Correlation among different targets. **Table S22**: Univariate Cox analysis of the association between markers and clinical outcome. **Table S23**: Univariate Cox analysis of the association between markers and clinical outcome in N0 and N+ group. **Table S24**: Univariate Cox analysis of the association between markers and clinical outcome in early-stage and advanced-stage groups. **Table S25**: Prognostic significance of biomarkers based on nodal status and stage of the disease. **Table S26**: Summary of genomewide differentially methylated regions as a function of deltathreshold. **Table S27**: Details for TaqMan qPCR CNV assays.

**Additional file 2. Figure S1**: Principal component analysisof methylation sites in the promoter region. **Figure S2**: Distribution of differential methylation at CpG sites in leukoplakia and OSCC. **Figure S3**: Volcano plots of −log10against the average methylation differenceshowing differentially hypo and hyper methylated promoters in OPL vs normal, tumor vs normal and tumor vs OPL. **Figure S4**: Distribution of differentially methylated promoters. **Figure S5**: Venn diagram. **Figure S6**: Boxplots showing differential promoter methylation between early and advanced-stage OSCC.

**Additional file 3**. Additional information.

## Data Availability

The dataset supporting the conclusions of this article is available in Gene Expression Omnibus (GEO); https:// www.ncbi.nlm.nih.gov/geo with accession number GSE204943. Token Code to access the GEO submission GSE204943-mnwxqokgdbkzhez.

## Abbreviations

AUC: Area under the curve
BH: Binyamini–Hochberg (method to correct for multiple testing)
CNA: Copy number alteration
DMP: Differentially methylated promoters
DSS: Disease-specific survival
FDR: False discovery rate
GBC: Gingivobuccal complex
GE: Gene expression
GEO: Gene expression omnibus
HPV: Humana papillomavirus
HR: Hazard ratio
LASSO: Least absolute shrinkage and selection operator
OPL: Oral premalignant lesions
OS: Overall survival
OSCC: Oral squamous cell carcinoma
RFS: Recurrence-free survival
ROC: Receiver operating characteristic
TSG: Tumor suppressor genes
