# Supplementary material for: Genome-wide DNA methylation profiling of HPV-negative leukoplakia and gingivobuccal complex cancers
Source: Clin Epigenetics. 2023 May 27;15:93. doi: 10.1186/s13148-023-01510-z (PMC10225107; doi:10.1186/s13148-023-01510-z)
Supplement: Supplementary file 2 — Additional file 2. Figure S1: Principal component analysisof methylation sites in the promoter region. Figure S2: Distribution of differential methylation at CpG sites in leukoplakia and OSCC. Figure S3: Volcano plots of −log10against the average methylation differenceshowing differentially hypo and hyper methylated promoters in OPL vs normal, tumor vs normal and tumor vs OPL. Figure S4: Distribution of differentially methylated promoters. Figure S5: Venn diagram. Figure S6: Boxplots showing differential promoter methylation between early and advanced-stage OSCC. [file 13148_2023_1510_MOESM2_ESM.pptx]

## Slide 1
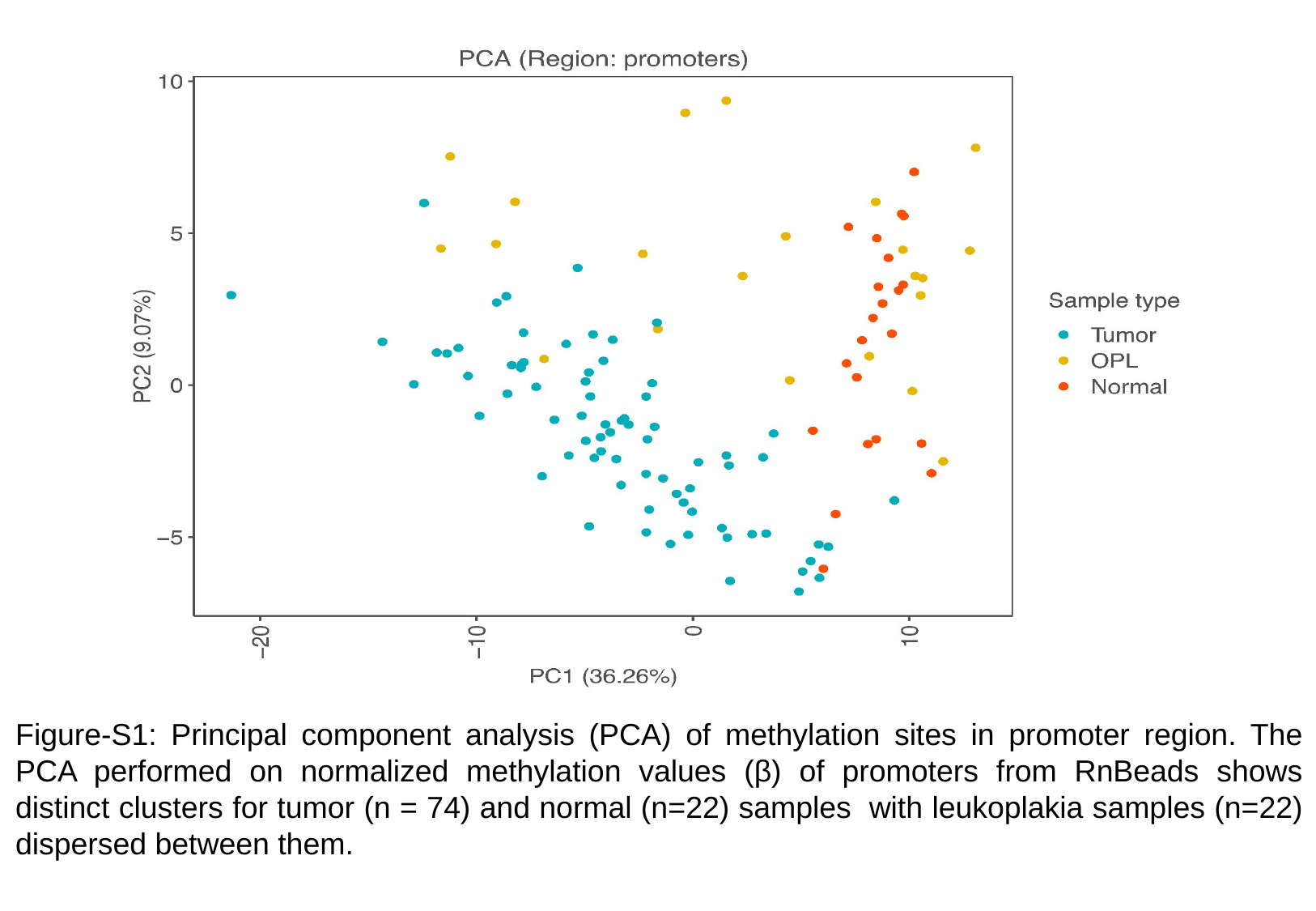

Figure-S1: Principal component analysis (PCA) of methylation sites in promoter region. The PCA performed on normalized methylation values (β) of promoters from RnBeads shows distinct clusters for tumor (n = 74) and normal (n=22) samples  with leukoplakia samples (n=22) dispersed between them.

## Slide 2
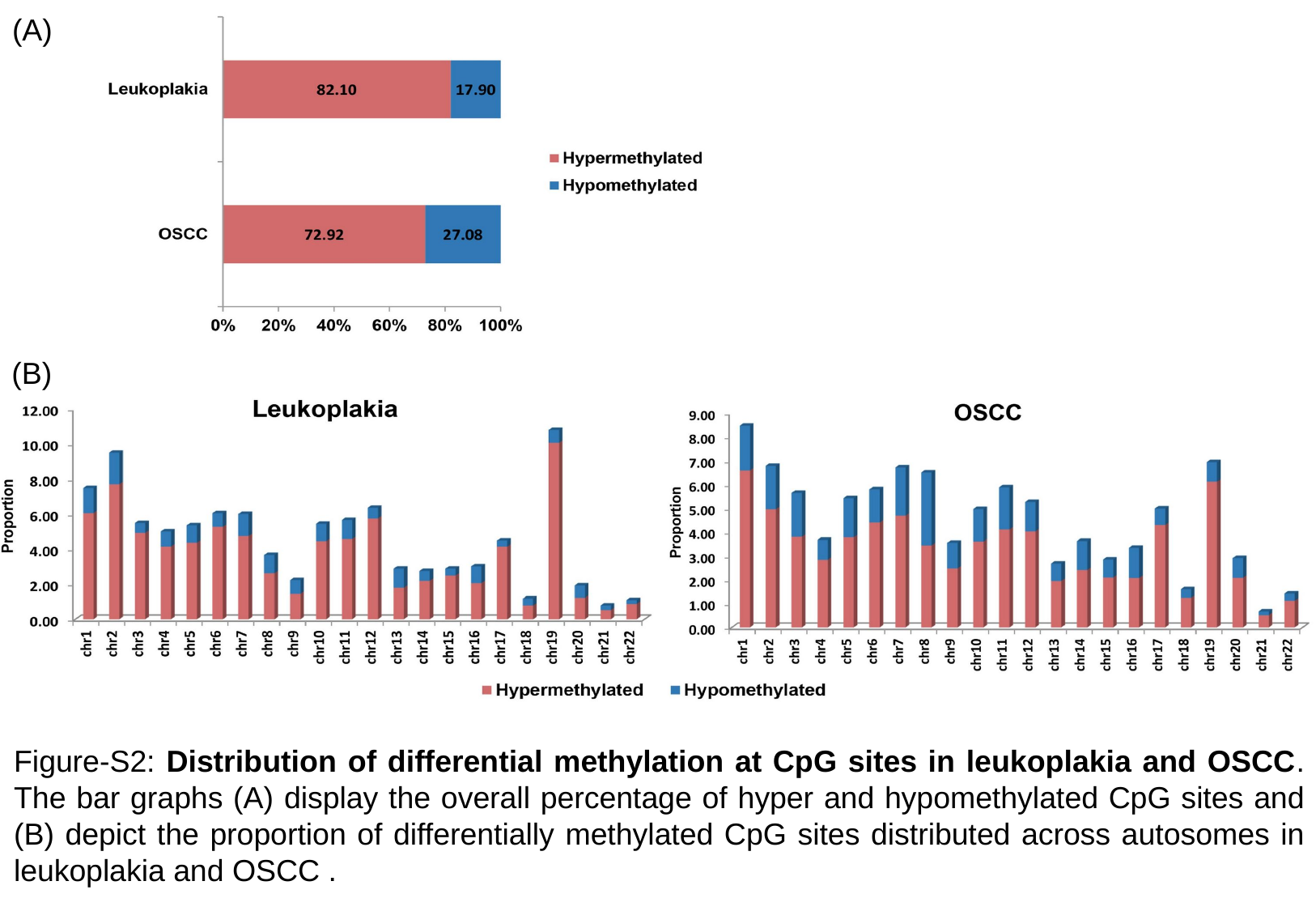

(A)
(B)
Figure-S2: Distribution of differential methylation at CpG sites in leukoplakia and OSCC. The bar graphs (A) display the overall percentage of hyper and hypomethylated CpG sites and (B) depict the proportion of differentially methylated CpG sites distributed across autosomes in leukoplakia and OSCC .

## Slide 3
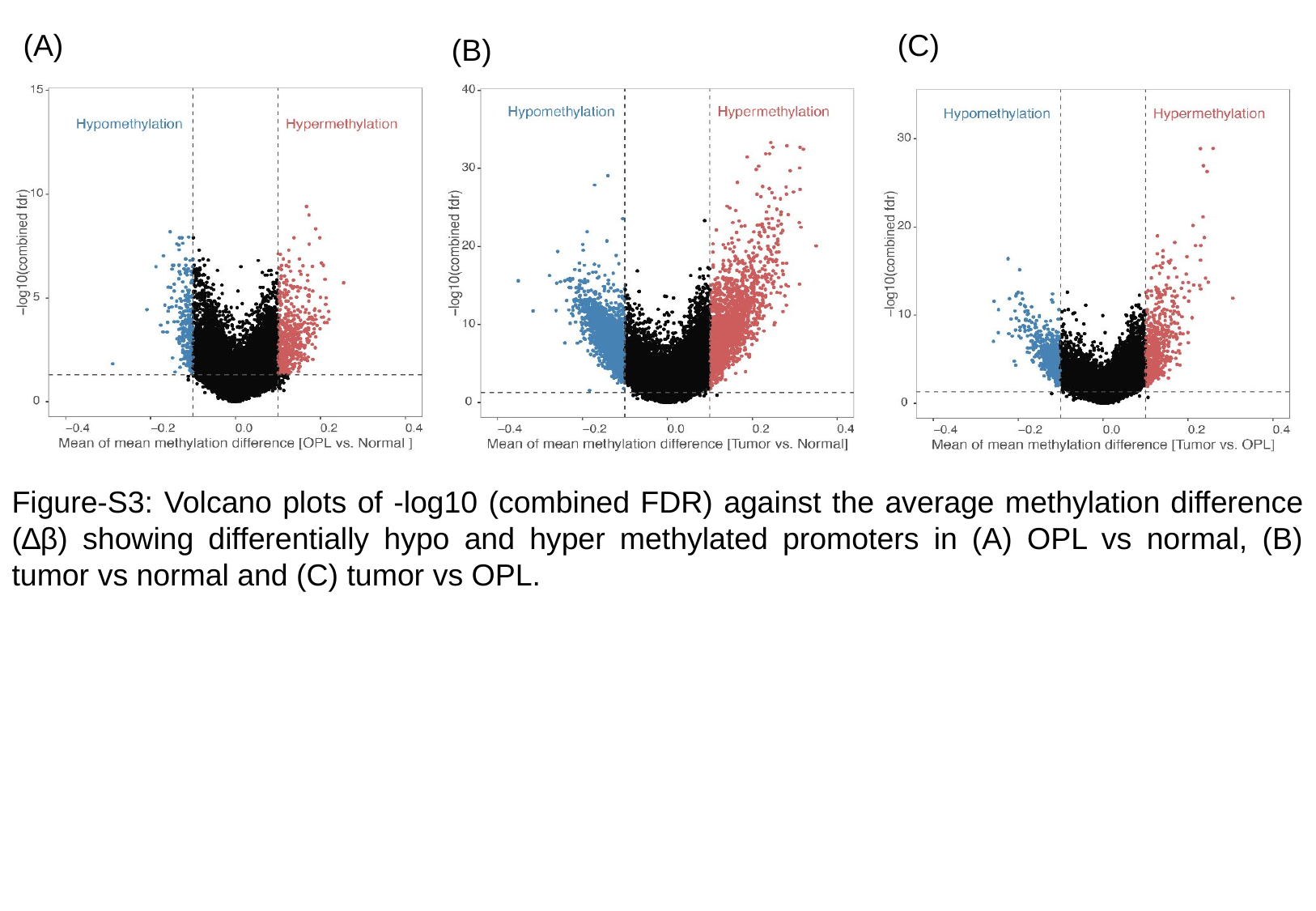

(A)
(C)
(B)
Figure-S3: Volcano plots of -log10 (combined FDR) against the average methylation difference (∆β) showing differentially hypo and hyper methylated promoters in (A) OPL vs normal, (B) tumor vs normal and (C) tumor vs OPL.

## Slide 4
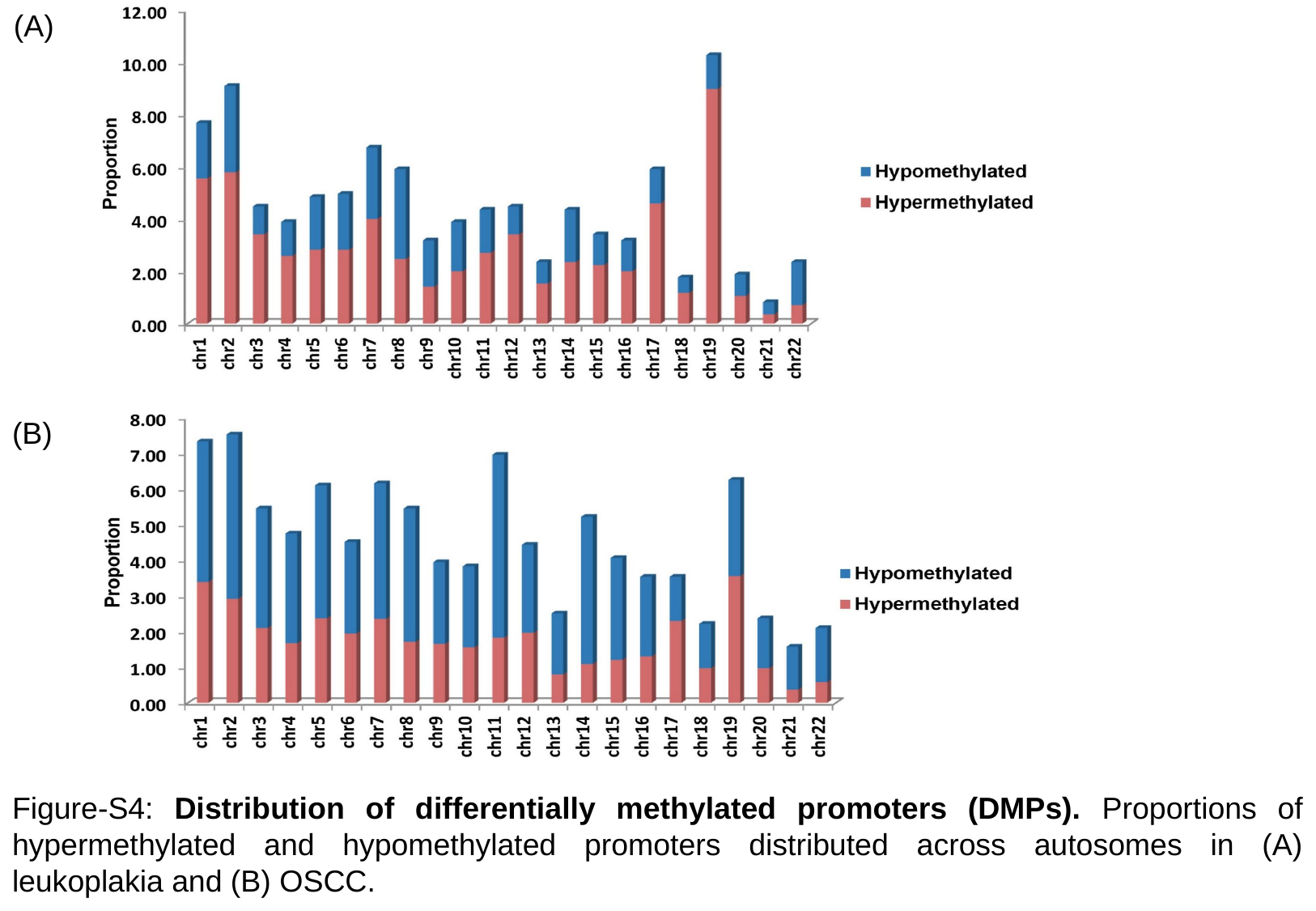

(A)
(B)
Figure-S4: Distribution of differentially methylated promoters (DMPs). Proportions of hypermethylated and hypomethylated promoters distributed across autosomes in (A) leukoplakia and (B) OSCC.

## Slide 5
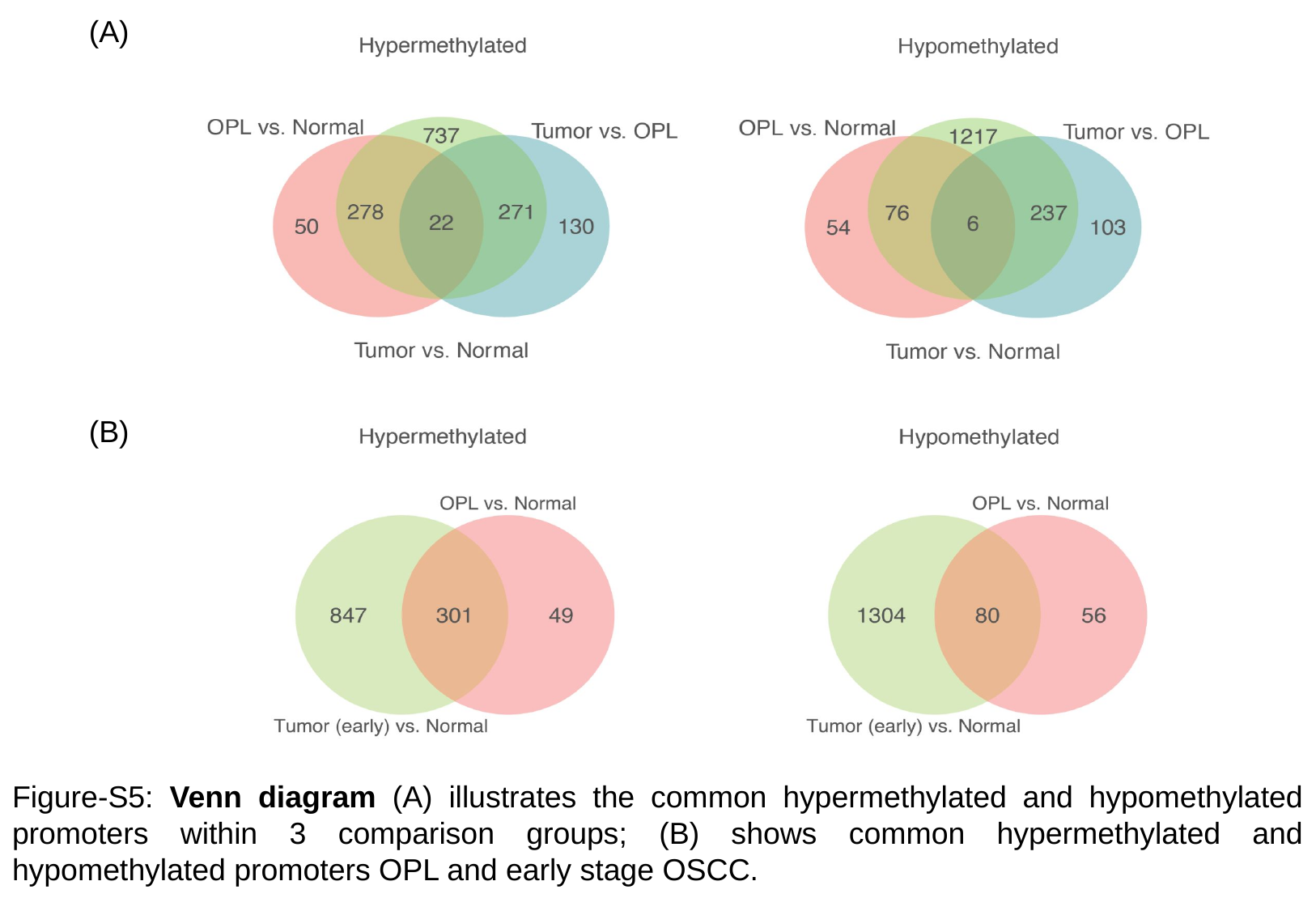

(A)
(B)
Figure-S5: Venn diagram (A) illustrates the common hypermethylated and hypomethylated promoters within 3 comparison groups; (B) shows common hypermethylated and hypomethylated promoters OPL and early stage OSCC.

## Slide 6
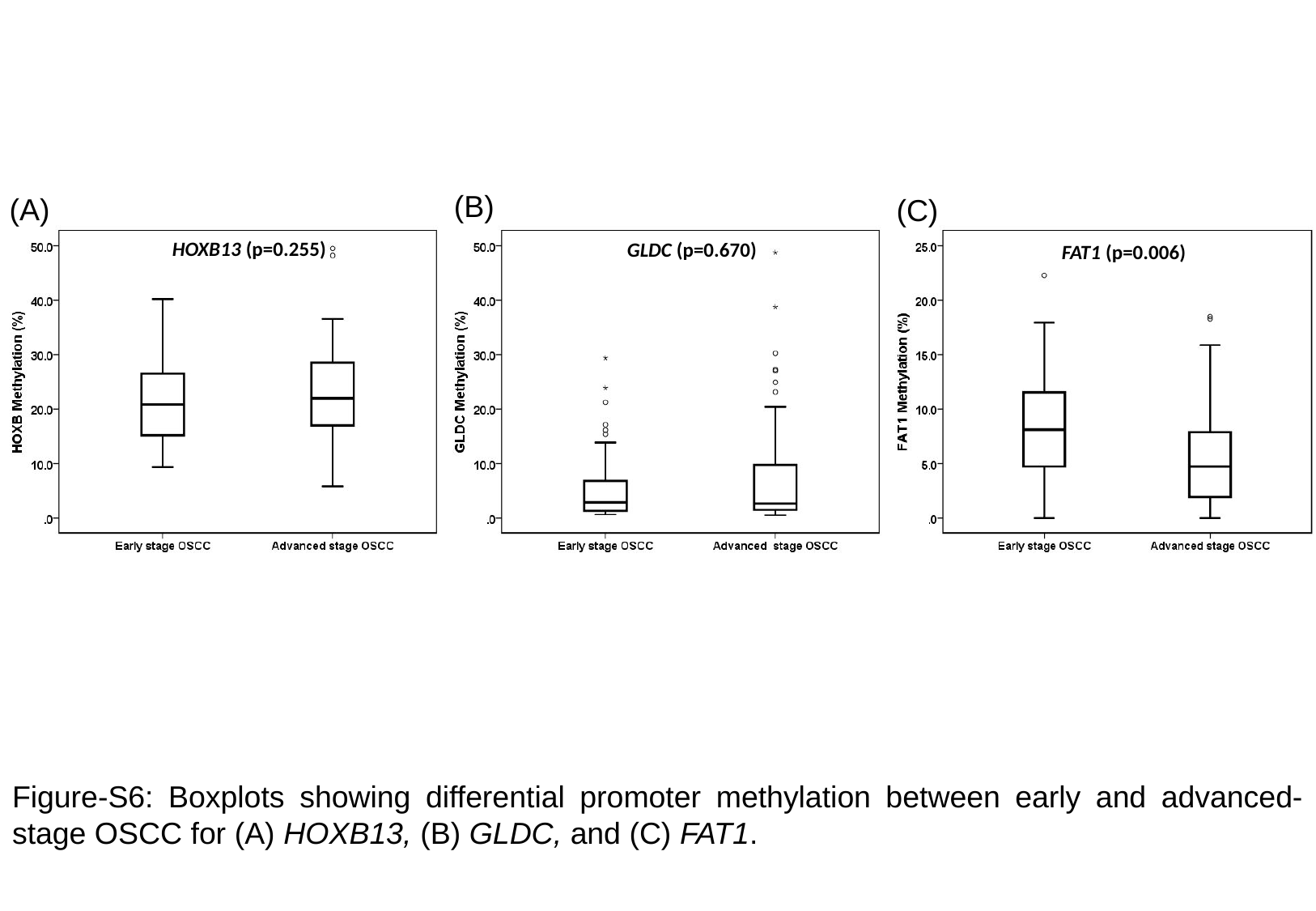

(B)
(A)
(C)
HOXB13 (p=0.255)
GLDC (p=0.670)
FAT1 (p=0.006)
Figure-S6: Boxplots showing differential promoter methylation between early and advanced-stage OSCC for (A) HOXB13, (B) GLDC, and (C) FAT1.
