# Supplementary material for: Genome-wide DNA methylation profiling of HPV-negative leukoplakia and gingivobuccal complex cancers
Source: Clin Epigenetics. 2023 May 27;15:93. doi: 10.1186/s13148-023-01510-z (PMC10225107; doi:10.1186/s13148-023-01510-z)
Supplement: Supplementary file 3 — Additional file 3. Additional information. [file 13148_2023_1510_MOESM3_ESM.docx]

**Supplementary information**

**Materials and methods:**

**Cryosectioning, DNA extraction and quality control:**

H&E staining was performed on 5µ tissue sections and the tumor content assessed by two pathologists independently. The samples having more than 70% of tumor content and hyperplastic and dysplastic in histology were subjected to DNA-RNA extraction and HPV detection. DNA was extracted from frozen tissues using AllPrep® DNA/RNA/miRNA Universal kit (Qiagen, Germany) by following the manufacturer’s instructions. Briefly, 25-30 mg of frozen tissue was grinded in mortar and pestle using liquid nitrogen and Buffer RLT Plus. The lysate was homogenised and passed through DNA and RNA columns for column purification. The DNA and RNA were eluted in 100ul and 50ul Elution buffers, respectively. The quality of extracted DNA was assessed by Qiaxpert (Qiagen, Hilden, Germany) and Qubit dsDNA BR Assay kit (Thermo Fisher Scientific, MA, USA). The integrity of DNA was checked by electrophoresis on 2% Agarose gel electrophoresis and amplifiability was determined by beta-globin PCR. The DNA samples were subjected to downstream analysis based on its quality and quantity.

**HPV analysis:**

The presence of HPV was detected by the protocol described in^1^. Nested PCR primers detect a wide range of high and low risk HPV subtypes by targeting the conserved region of the gene encoding the HPV viral capsid protein. Two types of primer sets were used; the first primer set MY09/MY11 yields a 450bp product and the second primer set GP5+/GP6+ gives a 150bp product. The HeLa cell line DNA, which is known to be infected with HPV, was used as a positive control.

**Bisulfite treatment and DNA methylation profiling:**

Genomic DNA was subjected to bisulfite treatment using Illumina recommended EZ DNA methylation Kit (Zymo Research, CA, USA) according to the manufacturer’s instruction. A total of 400ng genomic DNA was diluted with M-dilution buffer followed by addition of 100ul of CT Conversion reagent and kept it in the dark at 50^0^C for 12-16 hours. The converted DNA was purified using columns provided in the kit and eluted in 10ul of M-Elution Buffer. For long term storage, converted DNA was kept at -70^0^C.

The bisulfite treated DNA was denatured and isothermally amplified overnight. This whole-genome-amplified DNA was then fragmented using a controlled enzymatic process followed by precipitation and resuspension in a hybridization buffer. Eight samples were hybridised on each BeadChip in hybridization chambers and incubated for 18–20 hours at 48°C. After incubation, beadchip was washed and stained using labelled oligonucleotides. The single base extension using labelled oligos was detected using Illumina iScan System.

**Bisulfite pyrosequencing:**

Briefly, 600ng of genomic DNA from both normals and OSCC were subjected to bisulfite conversion using EpiTect Bisulfite Kit(Qiagen, Hilden, Germany) according to manufacturer's protocol and eluted in 30ul of Elution buffer(Qiagen) followed by quantification by NanoDrop. The PCR and sequencing primers were designed using the PyroMark Assay Design software, v2.0.1.15 (Qiagen) indicated in Additional file 1; Table-S18. Two ul of bisulfite treated DNA was amplified using PyroMark PCR kit according to the instructions. Each reaction was performed with 12.5ul 2X PyroMark PCR mastermix, 1X Forward and reverse primers in total 25ul reaction. All the PCR reactions were performed in a thermocycler (Veriti, 96 Well Thermal Cycler, Applied Biosystems, Foster City, California, USA) and PCR conditions were as follows: initial activation for 15 mins at 95^0^C, followed by 45 cycles of denaturation 30s at 94^0^C, annealing at various temperatures (Additional file 1; Table-S18.) for 30s, extension for 30s at 72^0^C, final extension for 10mins at 72^0^C. The PCR products were run on a 2% agarose gel to ensure PCR quality, correct product length and lack of contamination. DNA methylation levels at single CpG locus were determined by pyrosequencing using the Pyromark Q96ID Pyrosequencer (Qiagen) as per manufacturer’s instructions and using 1X sequencing primers.

**Copy number variation (CNV) analysis:**

All the reactions were performed four times using 10ng of DNA per reaction along with no template control in each batch. The VIC and TAMRA dye-labeled RNaseP is used as endogenous control and female pooled DNA as positive control. The RNaseP gene is located on chromosome 14q11.2 and has exact two copies of diploid genome. PCR conditions were as follows: 95 °C initial denaturation step for 10 minutes, 40 cycles at 95 °C for 15 seconds and 60 °C for 1 minute.

**Reference:**

1. Bhosale P G, Pandey M, Desai R S, Patil A, Kane S, Prabhash K, Mahimkar M B. Low prevalence of transcriptionally active human papilloma virus in Indian patients with HNSCC and leukoplakia*.* Oral Surg Oral Med Oral Pathol Oral Radiol 2016; 122: 609-618 e607.
